# Neuromodulation treats Chikungunya arthralgia: a randomized controlled trial

**DOI:** 10.1038/s41598-018-34514-4

**Published:** 2018-10-30

**Authors:** Edson Silva-Filho, Alexandre H. Okano, Edgard Morya, Jessica Albuquerque, Enio Cacho, Gozde Unal, Marom Bikson, Rodrigo Pegado

**Affiliations:** 1 Postgraduate Program in Rehabilitation Sciences, Federal University of Rio Grande do Norte, Santa Cruz, Brazil; 2 Center of Mathematics, Computation and Cognition, Universidade Federal do ABC, São Bernardo, São Paulo Brazil; 3 Edmond and Lily Safra International Institute of Neuroscience, Santos Dumont Institute, Macaiba, Brazil; 4 Department of Social Psychology, Federal University of Paraíba, João Pessoa, Brazil; 5 Department of Biomedical Engineering, The City College of The City University of New York, New York, USA

## Abstract

The Chikungunya (CHIK) virus is epidemic in Brazil, with 170,000 cases in the first half of 2016. More than 60% of patients present relapsing and remitting chronic arthralgia with debilitating pain lasting years. There are no specific therapeutic agents to treat and rehabilitee infected persons with CHIK. Persistent pain can lead to incapacitation, requiring long-term pharmacological treatment. Advances in non-pharmacological treatments are necessary to promote pain relief without side effects and to restore functionality. Clinical trials indicate transcranial direct current stimulation (tDCS) can treat a broad range of chronic pain disorders, including diffuse neuromuscular pain and arthralgia. Here, we demonstrate that the tDCS across the primary motor cortex significantly reduces pain in the chronic phase of CHIK. High-resolution computational model was created to analyze the cortical electric field generated during tDCS and a diffuse and clustered brain current flow including M1 ipsilateral and contralateral, left DLPFC, nucleus accumbens, and cingulate was found. Our findings suggest tDCS could be an effective, inexpensive and deployable therapy to areas lacking resources with a significant number of patients with chronic CHIK persistent pain.

## Introduction

The Chikungunya (CHIK) virus is a mosquito-transmitted alphavirus, first isolated in 1953 during an epidemic in Tanzania^1^. CHIK transmission occurs through the bite of an infected mosquito, Aedes aegypti or Aedes albopictu, from mother to fetus in the intrauterine perinatal period, through blood transfusion and sexual intercourse^2^. Mainly transmitted in urban areas, CHIK constitutes a significant public health problem in many countries, including in the Brazilian Northeast^3^. The burden of CHIK in Latin America is especially severe in Brazil with 170,000 cases in the first half of 2016, accounting for 94% of confirmed CHIK cases in the Americas^4^.

Patients infected with CHIK virus will develop persistent rheumatologic and general disabling symptoms such as joint pain, fever, asthenia, headache, retro-orbital pain, photosensitivity, muscular pains, back pain and tenosynovitis^1^. CHIK virus induced disease shares many similarities with illnesses caused by other arthritogenic alphaviruses^1^. Most patients develop severe and often debilitating polyarthralgia that is usually bilateral and symmetric, most commonly in ankles, wrists, and phalanges^1^. Although complete recovery of these symptoms occurs in some cases within four weeks, several individuals evolve into chronic disability pain for up to 6 years^5,6^. According to Silva, a large number of patients experience chronic musculoskeletal disorders and usually respond to some extent to analgesics, anti-inflammatory treatments, and physiotherapy^1^. In an attempt to reduce the physical symptoms caused by CHIK virus, several medications have been tested, such as chloroquine and meloxicam with limited effectiveness^7^.

The acute or chronic painful process generated by CHIK virus appears to be associated with the expression of specific metabolites in the human body influencing its severity after infection^8,9^. However, approximately 5% of patients meet the criteria for chronic inflammatory rheumatism, which may be destructive and deforming with severe pain symptoms and disability^1^. The current challenge for rehabilitation medicine is to provide an optimal treatment for CHIK rheumatic disorders and effectively disrupt the development course of CHIK symptoms.

Transcranial Direct Current Stimulation (tDCS) is a battery-powered non-invasive neuromodulation technique in which low amplitude direct current is conducted to the cortex^10^. Clinical trials indicate tDCS can treat a broad range of chronic pain disorders, including diffuse neuromuscular pain^11–13^. Studies with anodal stimulation of the primary motor cortex (M1) for the treatment of chronic pain syndromes including chronic back pain, fibromyalgia, trigeminal neuralgia, arthrosis and polyneuropathy demonstrated the effectiveness of tDCS on pain and mood symptoms^14,15^. Chronic pain may reflect not only local inflammation and peripheral sensitization, by having origins in the central nervous system reflecting maladaptive changes in brain excitability^16^. Active stimulation was found to be effective for reducing chronic rheumatic symptoms, with no adverse events and good tolerability^16^.

Anodal M1 and cathodal contra lateral supraorbital right (M1-SO) montages of tDCS may treat chronic pain by influencing pain matrix includes motor cortex and deep brain regions producing long-lasting changes in cortical connectivity or excitability^17,18^. tDCS thus offers promise as a novel neuromodulation approach for pain-related networks to alleviate CHIK rheumatic chronic symptoms, such as muscular pain and arthralgia. Moreover, tDCS is a low cost, safe, and mobile intervention that can be deployed on a large scale across locations such as health posts, clinics and in home-visit rehabilitation^19^.

The present study aim to investigate the hypothesis that tDCS would improve pain and functionality in subjects with chronicle CHIK arthralgia.

## Materials and Methods

The study was conducted in the Faculty of Health Science of Trairí, Federal University of Rio Grande do Norte. It was approved by the local institutional Ethical Committee (approval number 1.563.690) and conducted according to Declaration of Helsinki (1964), resolution No. 466/12 of the National Health Council, and the CONSORT recommendations^20^. The study was registered in clinicaltrials.gov on December 5, 2016 with the identifier NCT02993952. All participants provided written informed consent.

### Participants

The sample size was estimated based on previous studies of tDCS effect on rheumatic diseases^14,21,22^, and with significance of 0.05 and power of 0.80. Fregni suggested the mean reduction of 3 points in Visual Analogue Scale (VAS) for the experimental group was expected in contrast to no improvement in the sham group in rheumatic disease such as fibromialgia^22^. Thus, the sample size resulted in two groups of 10 participants.

All participants were included with previous serologic confirmation of Chikungunya (CHIK) virus infection based on CHIK virus IgG and IgM detected by direct ELISA/IgM/Euroimmun, according to the Central Laboratory (LACEN, Brazil). Paticipants from local communities of the Northeast of Brazil were recruited through advertisements in the electronic media and by health professionals from the communities. The inclusion criteria were positive laboratory tests for the CHIK virus for at least 6 months (chronic phase), preserved intellectual capacity determined by the Mini Mental State Examination (MMSE), physical capacity to do physical evaluation, and between 18 and 65 years old. The exclusion criteria were pain clearly related to any other etiology, such as dengue, zika, rheumatoid arthritis, gout, lupus, neurologic and muscular diseases, psychiatric illness, and history of drug abuse, signs or history of dizziness or epileptic disease, pregnancy, signs of severity and/or indication of hospitalization and metal implants in the head. Participants were not taking analgesic or other pain relieve medication during this study. All participants were women and only one men was included in Sham-tDCS.

#### Experimental design

**Figure 1:**
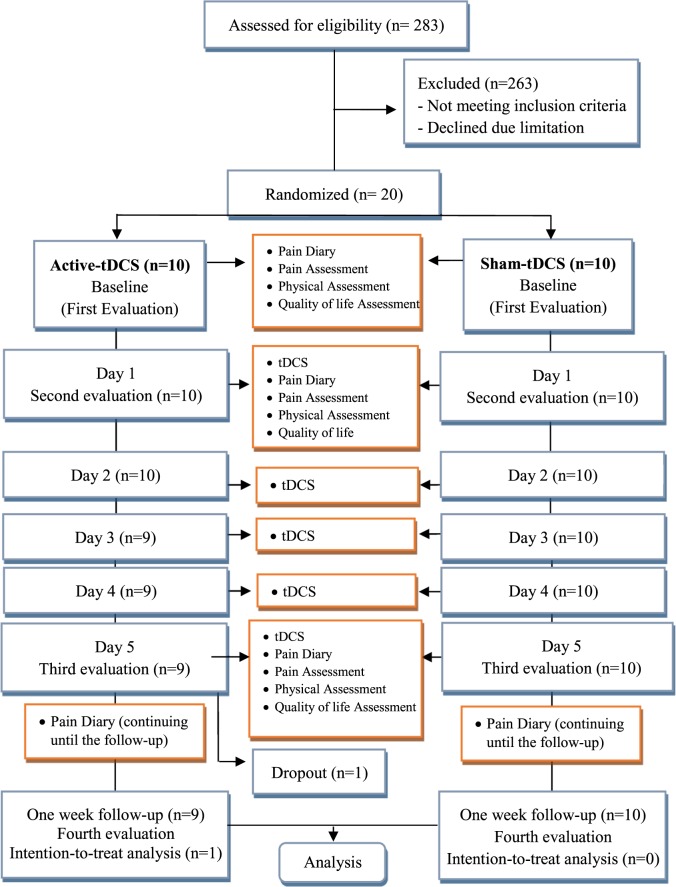
Flowchart summarizing the study. Twenty participants were randomized into two groups receiving active-tDCS or sham-tDCS on M1. Only one participant of active-tDCS group withdrew during the stimulation phase. Missing data were treated by intention-to-treat analysis. None of the participants received medication throughout the trial. Patients tolerated the tDCS treatments well, and no adverse effects occurred.

The flowchart illustrating the process of the study is shown in Fig. 1. This is a parallel, sham, randomized, double-blind trial with 1-week follow-up. Data were collected from December 2016 to March 2017. A total of 283 individuals were initially identified with laboratory tests for CHIK fever, but after applying the inclusion and exclusion criteria, 53 candidates with positive markers for CHIK fever were considered eligible for the study. Moreover, 33 individuals declined research participation due to the study protocol or the available time. Thus, 20 individuals were selected and randomized into 2 groups: active-tDCS group (n = 10) or sham-tDCS group (n = 10). One participant from the experimental group declined to continue because the improvement was not as expected, so intention-to-treat analysis was done in this group.

Participants were evaluated 1 week before the intervention (baseline), 1 day after the first intervention (day 1), after the last day of intervention (day 5), and 1 week after the last day of intervention. A random numerical sequence was generated (www.randomization.com) to assign each participant to either the active-tDCS group or the sham-tDCS group by an independent researcher not involved with either stimulation or assessments. Participants and researchers were blind to group allocation throughout the trial.

#### tDCS procedures

Participants were seated in a comfortable chair with back and arm support. tDCS was carried out with anode electrode on the left primary motor cortex (C3) and the cathode electrode on the right supraorbital region (Fp2) according to the international 10–20 EEG system (“M1-SO” assembly). Electrodes were encased in electrode sponges (7 × 5 cm) soaked in saline solution (150 mMols of NaCl diluted in water Milli-Q), and supported by a rubber band. Active tDCS was performed on 5 consecutive days of constant current of 2 mA for 20 min. A gradual current ramp-up and ramp-down of 30 s was used. Sham-tDCS was performed on 5 consecutive days with electrodes placed on the same position, and a constant current of 2 mA was delivered only for 30 s (10-s ramp-up) of the 20 min. Electrodes (anode and cathode) were connected to a battery (9 V) powered constant current stimulator with current verified by a precision digital multimeter (DT832, WeiHua Electronic Co., Ltd, China).

#### High-resolution computational model

Finite element models were created to analyze the cortical electric field generated during tDCS. High-resolution MRIs were segmented into seven tissue/material masks of varying conductivities through a combination of automated and manual tools. Computer generated models of electrodes, gel, and/or sponge pads were incorporated into the segmentation. Volume meshes were generated, boundary conditions were applied, and the Laplace equation $(\nabla \cdot (\sigma \nabla V)=0)$ was solved. The resulting cortical electric field was interpreted as a correlate for stimulation and modulation. Our results corroborate previous modeling studies, which show diffuse and clustered brain current flow that includes left motor regions and other regions implicated in the treatment of pain and other regions of interest. (in V/m: M1 ipsilateral 0.23, contralateral 0.15, left DLPFC 0.23, Nucleus accumbens 0.22, and cingulate 0.25).

#### Instruments and assessments

Socio-demographic data were collected, such as age, disease duration, pain intensity, pain location, marital status, and education. The measures of the outcomes were collected one week before the first simulation (baseline), after the first stimulation (day 1), after the last stimulation (day 5) and one week after the last stimulation (follow-up). Pain levels were evaluated with the self-reported Visual Analogue Scale (VAS), McGill Pain Questionnaire and Brief Pain Inventory (BPI) (short form).

#### Primary outcomes

VAS consisted of a gradient color-coded scale ranging from 0 (green; corresponding to complete absence of pain) to 10 (red; corresponding to worst imaginable pain) in intervals of 1 cm^23^. VAS was asked by a researcher after the first interview (one week before the first simulation - baseline), after day 1 and day 5 of tDCS and one week follow-up. In addition, participants recorded their daily pain intensity using VAS every night before sleeping. This pain diary completed 21 days of measurement. To analyze the homogeneity between groups, initial VAS pain levels were calculated for each patient by using the average of 1 week of pain diary reports.

#### Secondary outcomes

McGill Pain Questionnaire was used to characterize participants’ pain. It presents 20 items 10 sensorial, 5 affective, 1 evaluative and 4 miscellaneous. Each of these items displays from 2 to 5 options, and only one or none must be chosen^23^. The BPI (short form) was used to assess pain severity and impact in daily living activities. It presents 15 items, including 2 multi-item scales to measure pain and its impact on functionality and well-being^24^.

Participants performed a hand grip test (hydraulic hand dynamometer Saehan^**®**^ model SH5001) seated in a chair with armrests, shoulders at 0° of adduction, abduction, flexion, extension, internal and external rotation; elbow at 90° of flexion and wrist between 0° and 30° of extension. They were instructed to squeeze three times as hard as possible for 3 seconds and rest for 30 seconds between repetitions to have the arithmetic mean^25^. Strength of the lower limbs was evaluated by the 30-second chair stand test. A chair with 43 cm high, with backrest, without armrest and a stopwatch was used. Participants were instructed to keep their arms crossed on their chest, to stand and to sit with their backs resting on the backrest as fast as possible in 30 seconds. The score corresponds to the number of times performed to repeat the movement in 30 seconds^26^.

Upper limb flexion strength (dominant or more painful) was evaluated by the 30-second arm curl test. The test consisted of requiring the participants to hold a 2 kg weight and to perform full flexions and extensions as quickly as possible within 30 seconds. Participants could discontinue the test at any time, if necessary^27^.

To assess the flexibility of the lower limbs (posterior thigh muscles), a chair sit and reach test was performed. A chair with 43 cm high and 50 cm of backrest was used. Participants were instructed to sit on the edge of the chair with flat feet on the floor, knees and ankles at 90° flexion; then the dominant or painful leg was stretched (hip and knee) with the calcaneus supported on the floor and with the ankle flexed at 90°. With overlapping hands and with tips of the middle fingers even, participants tried to reach their toes and hold the reach for two seconds in three repetitions to get the arithmetic mean. A negative score was recorded if the middle fingers did not reach the toes, and a score was positive if the middle fingers were beyond the toes^27^.

Flexibility of the upper limbs was performed using the scratch flexibility test. The participants were instructed to pass one hand (dominant or more painful) over the shoulder to assess flexibility of the shoulder in flexion, abduction and external rotation and try to reach the other hand to assess extension, adduction and internal rotation on the center of the back. Three repetitions were made to obtain the arithmetic mean of the results. The scores were considered negative if there was any distance between the middle fingers, and positive if the middle fingers overlapped^27^.

A short form health survey (SF-36) was used^28^ to evaluate quality of life in the last four weeks. It consists of a 36 item questionnaire divided into 8 domains: functional capacity, limitation by physical aspects, pain, general health, vitality, social and emotional aspects and mental health. These domains have between 2 and 6 response options.

### Data analyses

Analyses were performed using the SPSS software (V.19.0, Chicago, USA) and Graph Pad Prism 5. Quantitative variables were expressed as means and standard deviations (SD). The Shapiro-Wilk and Levene’s test were applied to assess the normality of the distribution and homogeneity of variance of the data, respectively. Mauchly’s test of sphericity was used to validate the correlation of the repeated measures, and if the assumption of sphericity was violated, the Greenhouse-Geisser correction was applied. To compare age, time with CHIKV and VAS baseline between groups, an unpaired t-test or a Mann-Whitney test were used. The effects of stimulation on VAS were calculated using a mixed ANOVA model, in which the dependent variable was the level of pain, and the independent fixed variables were the treatment (baseline, day 1, day 5 and follow-up), the group of stimulation (active and sham) and the interaction term group vs. time. To determine the difference between groups at each category of time and vice versa it was performed three separate between-subjects ANOVAs. When appropriate, post-hoc comparisons were carried out using Bonferroni correction for multiple comparisons.

For other outcome variables (diary of pain, McGill questionnaire, Pain Inventory, physical tests and SF36), a non-parametric Friedman test was used. Missing data were treated by intention-to-treat analysis, taking into account the method of the last observation carried forward. Partial η^2^ were calculated as measures of effect size in the ANOVA results (main effects and interaction effects). Partial η^2^ was used to calculate the effect size, where η^2^ = 0.01 was considered small, η^2^ = 0.06 moderate and η^2^ = 0.14 large effect. Statistical significance was set at *p* ≤ 0.05.

## Results

Twenty patients in the chronic phase of CHIK were randomized into two groups receiving active-tDCS or sham-tDCS with a “M1-SO” assembly. Nineteen individuals completed the intervention period and there was one withdrawal in active-tDCS group. No differences in age (p = 0.969), time with CHIKV (p = 0.806) and VAS baseline (p = 0.257) were found between groups. There were no socio-demographic differences between groups (Table 1).

**Table 1:** Socio-demographic and pain characteristics.

Clinical and demographic data	Active-tDCS	Sham-tDCS	p value
Mean age ± SD (range)	46.1 ± (16.0)	44.1 ± (13.5)	0.969^α^
Time with CHIKV	10.2	10.4	0.806^β^
VAS baseline SD (range)	5.37 ± (2.4)	4.08 ± (3.02)	0.257^β^
Pain locations			0.865^γ^
More than 1 pain region	2	2	
Head/neck	0	0	
Thorax/abdomen	0	0	
Back	0	0	
Upper Limbs	3	2	
Lower Limbs	5	6	
Marital status			0.494^γ^
Married	7	8	
Never Married	2	1	
Widowed	1	0	
Divorced	0	1	
Education			0.779^γ^
Elementary (incomplete)	1	1	
Elementary	3	3	
Secondary	6	5	
University	0	1	

SD = Standard Deviation; CHIK = Chikungunya virus; VAS = Visual Analogue Scale. Age described in years with mean and standard deviation. Time with CHIK fever described in months. VAS baseline described with the mean of seven days before intervention using VAS diary. αCalculated using Mann Whitney. ^β^Calculated using unpaired t test. ^γ^Calculated using chi-square test.

**Figure 2:**
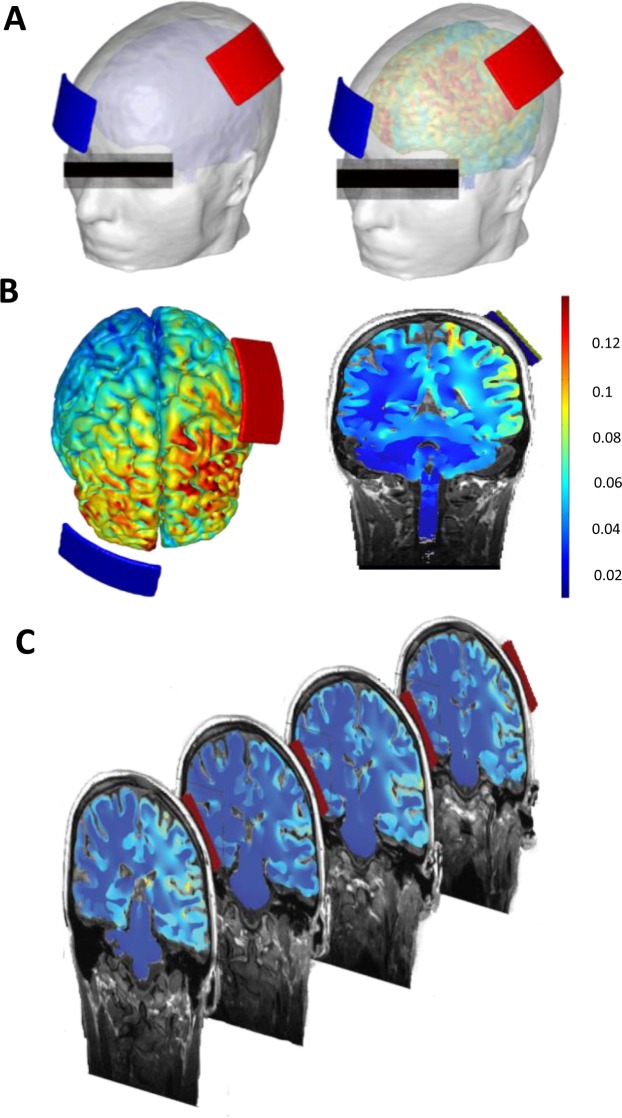
(**A**) High-resolution computational model simulation of brain current flow during tDCS. The “M1-SO” assembly was simulated by sponge with anode positioned vertically over 10–20, location C3, and cathode positioned horizontally on the contralateral-supraorbital, approximately over 10–20, location Fp2. (**B**) Current flow patterns on a slice (A/m^2^). The model predicted electric field maps generated in outer cortical regions. Our results show diffuse and clustered brain current flow that includes left motor regions, as well as other regions implicated in treatment of pain and other regions of interest. (in V/m: M1 ipsilateral 0.23, contralateral 0.15, left DLPFC 0.23, nucleus accumbens 0.22, and cingulate 0.25). (**C**) Consecutive coronal slices and brain current flow.

Active-tDCS simulation of brain current flow (M1-SO) is predicted to produce electric fields across the brain pain neuromatrix regions, including motor cortex, DLPFC, nucleus accumbens and cingulate (Fig. 2B).

**Figure 3:**
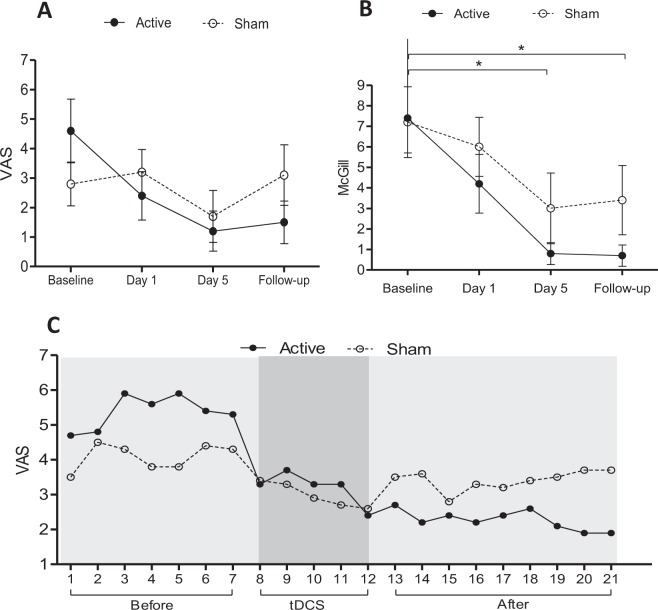
(**A**) Mean visual analog scale (VAS) before treatment (baseline), on days 1 and 5 (during treatment) and follow-up. Error bars denote SEM. (**B**) Mean McGill score before treatment (baseline), on days 1 and 5 (during treatment) and follow-up. *p < 0.05. (**C**) Mean daily VAS pain recording. tDCS: describes the five days of treatment. After: denotes the follow-up period. A statistically significant decrease in pain was found only in Active-tDCS (p < 0.05, Friedman).

Pain was assessed using VAS at four intervals (baseline, day 1, day 5 and 1 week follow-up). A mixed ANOVA model indicated significant interaction between the group and time on pain evaluated using VAS, F(3.54) = 3.815, p = 0.01, partial η^2^ = 0.175. Similarly, there were significant main effects of time, F(3.54) = 17.712, p = 0.001, partial η^2^ = 0.956. It was performed the simple main effect for group and time for testing differences in VAS between groups at each category of the within-subjects factor using three separate one-way ANOVAs. There was no statistically significant simple main effect difference in VAS between groups at the baseline, *F*(1,18) = 1.894, *p* = 0.18, partial η^2^ = 0.09; day 1 *F*(1,18) = 3.200, *p* = 0.48, partial η^2^ = 0.02; day 5* F*(1,18) = 1.250, *p* = 0.65, partial η^2^ = 0.01 and follow-up *F*(1,18) = 12.800, *p* = 0.21, partial η^2^ = 0.08. There was a statistically significant simple main effect of time on VAS for active-group F(3,27) = 7.300, p = 0.008, partial η2 = 0.44. No significant effect of time was showed for sham-group F(3,27) = 2.019, p = 0.135, partial η^2^ = 0.18 (Fig. 3A).

McGill’s pain questionnaire showed significant effect of Active-tDCS during the intervention, χ2(4) = 18.113, p = 0.001 (Fig. 3B), while there was no effect of Sham-tDCS, χ2(4) = 4.514, p = 0.341. Post hoc analysis in the Active-tDCS group indicated significant differences between baseline vs day 5 (p = 0.001) and follow-up (p = 0.01). Participants recorded daily VAS pain sensation during 21 days (7 days before intervention, during the intervention and 7 days after). Friedman test showed a significant difference only for the Active-tDCS group (p < 0.0001) (Sham-tDCS group p = 0.417) (Fig. 3C). Brief pain inventory showed significant results in intragroup analysis for Active-tDCS in the variables: worst pain of the last 24hs, average of pain, pain at the moment, general activity and normal work, while for sham-tDCS there was no significant difference (Table 2).

**Table 2:** Intra-group analysis of the Brief Pain Inventory at Baseline, Day 1, Day 5 and Follow-up.

Pain Interferes	Active-tDCS				p value	Sham-tDCS				p value
	Baseline	Day1	Day5	Follow-up		Baseline	Day1	Day5	Follow-up	
Worst pain in last 24 hs	6 (3.75–8)	5 (1.5–7.25)	4 (0–7)	2 (0–5.5)	0.03*	4 (0–6.5)	6.5 (2.2–8)	2 (0–6)	3.5 (0–5.2)	0.34
Least pain in last 24 hs	2.5 (1.5–4)	2 (0–4.5)	1.5 (0–4)	0 (0–3.2)	0.13	1 (0–3.2)	4 (0.7–5.2)	0.5 (0–3)	0 (0–3.2)	0.08
Pain average	5.5 (2.2–6.5)	4 (1.5–6.2)	3 (0–5)	1 (0–4.5)	0.02*	2.5 (0–5.2)	5 (1.5–6.2)	1.5 (0–4.7)	3 (0–5.2)	0.28
Pain at the moment	5 (1.5–6)	2.5 (0–5.2)	0 (0–2.5)	0 (0–2.5)	0.001*	2 (0–5.2)	3.5 (1.5–6)	0.5 (0–3)	1 (0–5.2)	0.18
General Activity	4.5 (0–5.7)	5 (0–7)	0 (0–6)	0 (0–4.5)	0.05*	4 (0–5.7)	3 (0–7)	1 (0–3.7)	1 (0–2.5)	0.28
Mood	0 (0–4.2)	2 (0–6.2)	0 (0–5)	0 (0–3.5)	0.25	0.5 (0–5.2)	1 (0–4.7)	0.5 (0–4.2)	0 (0–2.2)	0.37
Walking ability	1.5 (0–5.2)	1.5 (0–6.2)	0 (0–3.5)	0 (0–1.2)	0.34	2.5 (0–5.2)	2.5 (0–5.7)	0.5 (0–6)	1 (0–3.2)	0.49
Normal Work	4.5 (1.5–6)	2.5 (0–6.2)	0 (0–3.5)	0 (0–1.2)	0.04*	4 (0–6.5)	4.5 (0–8)	1.5 (0–3.5)	2 (0–3.2)	0.06
Relationship	0 (0–0.7)	0 (0–2.7)	0 (0–1)	0 (0–0)	0.57	0 (0–4.2)	1.5 (0–5.2)	0 (0–1.7)	0 (0–1.7)	0.09
Sleep	0 (0–4)	0 (0–5)	0 (0–1.5)	0 (0–0)	0.3	0 (0–1)	0 (0–2)	0 (0–0)	0 (0–0)	0.28
Enjoyment of life	0 (0–0.5)	0 (0–1.2)	0 (0–0)	0 (0–0)	0.3	0 (0–4.2)	1 (0–8)	0 (0–1.5)	0 (0–0)	0.16

The results were expressed as median, 25^th^ percentile and 75^th^ percentile. BPI: Brief Pain Inventory; tDCS: transcranial direct current stimulation. *Indicates statistically significant (p < 0.05) when comparing Baseline, Day 1, Day 5 and Follow-up using Friedman test.

Hand grip strength test showed a trend to improve in Active-tDCS group (χ2(3) = 6.704, p = 0.08; Sham-tDCS: χ2(3) = 2.526, p = 0.47). Chair stand, Arm-curl, Chair-sit and reach showed no difference between time and intervention: Active-tDCS (χ2(3) = 4.225, p = 0.23); Sham-tDCS (χ2(3) = 6.148, p = 0.10); Active-tDCS (χ2(3) = 4.014, p = 0.26); Sham-tDCS (χ2(3) = 1.479, p = 0.68); Active-tDCS: (χ2(3) = 6.750, p = 0.08); Sham-tDCS: (χ2(3) = 0.273, p = 0.96), respectively. Back scratch test showed significant difference in the Active-tDCS group (χ2(3) = 15.289, p = 0.002). Baseline back scratch indicated significant difference with day 5 (p = 0.01) and follow-up (p = 0.001). However, Sham-tDCS was also significant (χ2(3) = 11.762, p = 0.008). SF-36 questionnaire showed non-significant difference in both Active-tDCS groups (χ2(3) = 5.055, p = 0.16) and Sham-tDCS (χ2(3) = 3.935, p = 0.26). These tests aimed to evaluate the functionality as function of deficits in resistance, power, mobility and flexibility. These physical fitness tests are simple, quick and economical evaluation, especially in primary care settings and rheumatologic diseases^29^.

## Discussion

Here we showed significant benefits of tDCS on pain sensation following five consecutively days of M1-SO tDCS at 2 mA for 20 min. Our results suggest tDCS could be a critical cost-effective approach for a non-pharmacological strategy to pain relief in a large number of people afflicted by Brazilian CHIK virus epidemic. tDCS has not been previously evaluated to treat CHIK, and the ineffectiveness of the rehabilitation methods to treat the chronic phase of CHIK inspired us to conduct the first clinical trial with tDCS in this population. This preliminary study indicates that several aspects of pain were already ameliorated when M1-SO tDCS was applied for only 5 consecutive days. These results encourage further clinical trials, including long term tDCS treatment (e.g. 10 or 20 sessions) to enhance efficacy. Indeed, CHIK very often results in severe chronic arthralgia and/or arthritis, which can last months to years following the initial infection^30,31^.

Several studies suggest that chronic pain conditions, such as primary dysmenorrhea, irritable bowel syndrome, interstitial cystitis, fibromyalgia and low back pain, may develop an adaptive neuroplasticity and functional reorganization in pain-related areas^32,33^. Thus, these areas in the pain neuromatrix (medial prefrontal cortex, posterior cingulate cortex and insula) may exhibit abnormal functional and structural changes^32,33^. Therefore tDCS can modulate the neuromatrix and provide a significant reduction of pain in patients with chronic pain conditions^14^. One physiological explanation would be that the tDCS may change maladaptive plasticity both in originally impacted and secondary brain regions in chronic pain syndromes^12,15,34^. Consistent with this hypothesis, the pain treatment related to CHIK virus in this trial was performed during the chronic phase (after 6 months) and significant improvements were shown for pain and its impacts on daily activities according to BPI. Nonetheless, tDCS effect must be investigated during acute stage, where the maladaptive plasticity might be more susceptible to change, and to increase the benefit. We hypothesize that the association between chronic pain in the CHIK virus and brain reorganization could also exist in others chronic pain syndromes, and tDCS may show similar beneficial results. Mechanism of tDCS action should prevent or revert the ongoing maladaptive plasticity within the pain matrix^35^.

Moreover, repetitive transcranial magnetic stimulation (rTMS) on M1 was associated with improvement in quality of life and pain in fibromyalgia, chronic pelvic pain and neuropathic pain^36–38^. These evidences support the use of neuromodulation for analgesic effects in chikungunya. While, the M1/SO is the most common tDCS electrode montage for pain^11,14^, Several meta-analysis suggest an evidence for the effectiveness of anodal M1 stimulation over pain relief^34^, and others tDCS montage such as dorsolateral prefrontal cortex (DLPFC/SO), primary sensory cortex (S1/SO) or cerebellar (cerebellum/Shoulder) could be used for pain relief^34,39^.

VAS is the most common method of pain evaluation in clinical trials with tDCS and TMS^14^, and several adult population, including rheumatic diseases^40^. Our results showed a reduction over a 30% in VAS, which can be clinically relevant according to Klein at el^41^. The absence of differences in physical outcomes between the active and sham group could be related to: (1) fitness tests to discriminate specific physical function for CHIK; (2) number of participants (clinical trial power); (3) need to further optimized dose including more sessions. M1-SO tDCS for fibromyalgia showed reduction on pain scores, anxiety, depression and improvement in sleep quality and cognitive performance, but no difference in physical performance^14^. Therefore, there remains the need of specific physical performance test for CHIK in chronic phase, especially in arthritic symptoms.

According to SF-36 no significant improvement of quality of life was found, and similar studies using the same intervention, but for chronic abdominal pain also showed the same results^42^. This may reflect the limited number of session and/or the period for participants’ evaluation was insufficient to induce changes in normal routine. In addition, quality of life aspects in CHIK are multifocal and include not only joint pain, but also mental health, emotional role and social functioning^5^. It can be suggested that future intervention studies with neuromodulation and CHIK should observe not only rheumatic symptoms, but also the social and emotional context of the participants. BPI could be used to evaluate intensity, location and pain effect on the quality of life in patients with CHIK according to Andrade *et al*.^40^. Moreover, BPI showed significant improvement in normal work and general activities, suggesting that tDCS could improve the functional status of daily activities.

Marimoutou *et al*. showed a significant self-reported over morbidity and impaired quality of life persisting in CHIK patients for 6 years after infection^5^, while in our study the average was 10 months of CHIK infection. Furthermore, chronic CHIK arthralgia has a large psychological impact, frequent depressive moods and social disabilities^5^. These clinical findings influence quality of life and functionality. The M1-SO tDCS used in this trial is note the best assemble to treat depression and others montages should be tested.

In countries that have already suffered large CHIK outbreaks, attention is focused on the long-term burden of persistent arthralgia^5^. Understanding new rehabilitation approaches will greatly contribute to future decision-making on appropriate allocation of resources for public health care and research.

Our results provide evidence that tDCS reduces the pain levels with clinically significant changes for patients with the CHIK virus. Future investigations involving additional numbers of sessions of tDCS in CHIK patients could prove more effective not only in pain relief, but also in cognitive function, physical activity assessment, quality of life and general functionality. The tDCS treatment could be useful, inexpensive and attractive to areas lacking resources and with a high number of patients with CHIK symptoms. This study brings a novel approach to the non-pharmacological treatment for CHIK fever with tDCS.

## Data Availability

The datasets generated during and/or analysed during the current study are available from the corresponding author on reasonable request.
